# Frequency modulation of cortical rhythmicity governs behavioral variability, excitability and synchrony of neurons in the visual cortex

**DOI:** 10.1038/s41598-022-25264-5

**Published:** 2022-12-03

**Authors:** Mohammad Bagher Khamechian, Mohammad Reza Daliri

**Affiliations:** 1 Neuroscience and Neuroengineering Research Laboratory, Biomedical Engineering Department, School of Electrical Engineering, Iran University of Science and Technology, Tehran, 16846-13114 Iran; 2 Cognitive Neurobiology Laboratory, School of Cognitive Sciences, Institute for Research in Fundamental Sciences, Tehran, Iran

**Keywords:** Attention, Perception, Visual system

## Abstract

Research in cognitive neuroscience has renewed the idea that brain oscillations are a core organization implicated in fundamental brain functions. Growing evidence reveals that the characteristic features of these oscillations, including power, phase and frequency, are highly non-stationary, fluctuating alongside alternations in sensation, cognition and behavior. However, there is little consensus on the functional implications of the instantaneous frequency variation in cortical excitability and concomitant behavior. Here, we capitalized on intracortical electrophysiology in the macaque monkey’s visual area MT performing a visuospatial discrimination task with visual cues. We observed that the instantaneous frequency of the theta–alpha oscillations (4–13 Hz) is modulated among specific neurons whose RFs overlap with the cued stimulus location. Interestingly, we found that such frequency modulation is causally correlated with MT excitability at both scales of individual and ensemble of neurons. Moreover, studying the functional relevance of frequency variations indicated that the average theta–alpha frequencies foreshadow the monkey’s reaction time. Our results also revealed that the neural synchronization strength alters with the average frequency shift in theta–alpha oscillations, suggesting frequency modulation is critical for mutually adjusting MTs’ rhythms. Overall, our findings propose that theta–alpha frequency variations modulate MT’s excitability, regulate mutual neurons’ rhythmicity and indicate variability in behavior.

## Introduction

Recording electrical fields in cortical neurons, denoted as neural field potentials, provides rich information about the physiological, behavioral and cognition profiles of the brain. One conspicuous feature of such recordings is sustained-rhythmic activity, referred to as neural oscillations, which appears at various levels of the neural system^1^. Neural oscillations representing the brain's chaotic activity are a critical reflection of neural working mechanisms. Owing to their importance and ubiquity, they have been widely studied across different species in neurophysiological studies^2–4^. Accordingly, theoretical and physiological evidence suggests that oscillations functionally shape spatiotemporal dynamics of network activities in the brain^5–9^. Yet, neural oscillations are thought to rely on population synchrony^10^, reflecting cyclic variations in the excitability of neuronal ensembles^11^.

By and large, most studies focus on characteristic features of oscillations such as phase and amplitude dynamics, which can be modulated due to changes either in the brain’s internal state (attention or other cognitive abilities)^12–17^ or through external stimulation^18–21^. Nonetheless, growing evidence emphasizes the importance of variations in the frequency of the oscillations within a specific band for conducting behavior and neural processing^22–30^. For example, several findings have highlighted the relevance of fluctuation in theta–alpha frequencies to the modulation in temporal resolution of visual perception^23,24^, cortical excitability^22,26^, the temporal resolution of the binding window^31^, working memory capacity^32^, spatial and non-spatial cognitive processing^33^, task difficulty^27^, sensory stimulation and task engagement^22,26,27,34,35^. Basically, the frequency variability is thought to constitute the basis of an adaptive and self-regulated mechanism reflecting the activation level of neural populations^29^.

Surprisingly, recent findings imply that spatial attention is a critical cognitive function operating periodically^36–41^, imposing rhythmicity among visual sensory^42,43^ and frontal^44,45^ cortices to processing visual information in discrete temporal windows over time. A growing literature has also underscored that such attentional temporal windows are clocked at frequencies lower than 20Hz^38,41,46–48^, entrained by brain oscillations at corresponding frequencies^44,45,49,50^. These observations imply that the brain engages oscillations to sample sensory information rhythmically^41,51–54^. Recent findings suggest that such rhythmic sampling is performed in precise moments with the high-excitability phase of the neural oscillation^7,55^.

Interestingly, when two ensembles of neurons synchronously oscillate in phase, they can functionally facilitate the transmission of information to a target ensemble of neurons in downstream regions^6,7^. Even negligible deviations in the frequency of oscillations in one group with respect to the other may dramatically alter the phase synchrony and also the subsequent dynamic process underpinning routing information^28^. These arguments lead to doubts about the functionality of phase synchronization that reflects a stationary regime of synchrony, assuming that the underpinning oscillatory dynamics remain stable at a particular fixed phase relation and common frequency. However, a recent study of neurons in monkeys' visual area V1 demonstrated that stronger non-stationary frequency modulations result in more robust synchronization and, therefore, more reliable phase coordination^56^. This finding, indeed, uncovers the significance of instantaneous frequency modulations for regulating phase relations and synchronization of neurons in brain networks. From a dynamical system outlook, groups of neurons underlying non-stationary synchronization adjust their rhythms mutually through phase shifts and altering the instantaneous frequency^57,58^.

Despite the significance of frequency variations in the brain’s functions, the following questions remained elusive; to what extent can the oscillation frequency of neurons be modulated in response to varying stimulus properties in the sensory visual cortex? If such frequency modulation happens, what is the associated functional and behavioral significance? How do underlying oscillation frequency changes influence the spiking outputs at the levels of single and ensembles of neurons? Is there any causal relationship between input frequency intensity (in Hertz) and output firing of neurons? Given frequency variations underlying non-stationary neural synchronization in the primary visual cortex, how are these two parameters connected in extrastriate areas?

To examine these questions, we recorded intracortical data from a monkey’s visual area MT performing a visuospatial change detection task. The use of this task allowed us to explore whether frequency variations are tied explicitly to the top-down processing of spatial attention. Here, we adopt a new approach based on windowed harmonic wavelet transform^59^ to measure the instantaneous frequency of brain oscillations. Briefly, the harmonic wavelet transform (HWT), similar to the ordinary discrete wavelet transform, uses the fast Fourier transform algorithm to conduct a multi-resolution analysis for a non-stationary signal. A particular advantage of the HWT is that it integrates the benefits of the continuous wavelet transform and the short-time Fourier transform to estimate the signal phase without using band-pass filters^59^. We hypothesize that frequency modulations of cortical rhythms, projecting top-down influences, can potentially impact the brain’s functions at various levels of the neural system, from simple activity of single units (e.g. changes in the firing rate levels) to the complex processing of networks (e.g. modulations of synchronization) in MT area.

## Methods

### Animal welfare

All Animal procedures of this study carried out at the German Primate Center in Gottingen, Germany, were approved by the responsible regional government office [Niedersaechsisches Landesamt fuer Verbraucherschutz und Lebensmittelsicherheit (LAVES)], under permit numbers 33.42502/08–07.02 and 33.14.42502–04-064/07. Moreover, whole experiments and analysis methods declared in the manuscript follow the recommendations in the ARRIVE guidelines. We also confirm that all methods were performed in accordance with the relevant guidelines and regulations. For more information about the experimental procedure, details of surgical techniques, and methods used for animal training, see^6^.

### Experimental task and electrophysiological recording

**Figure 1:**
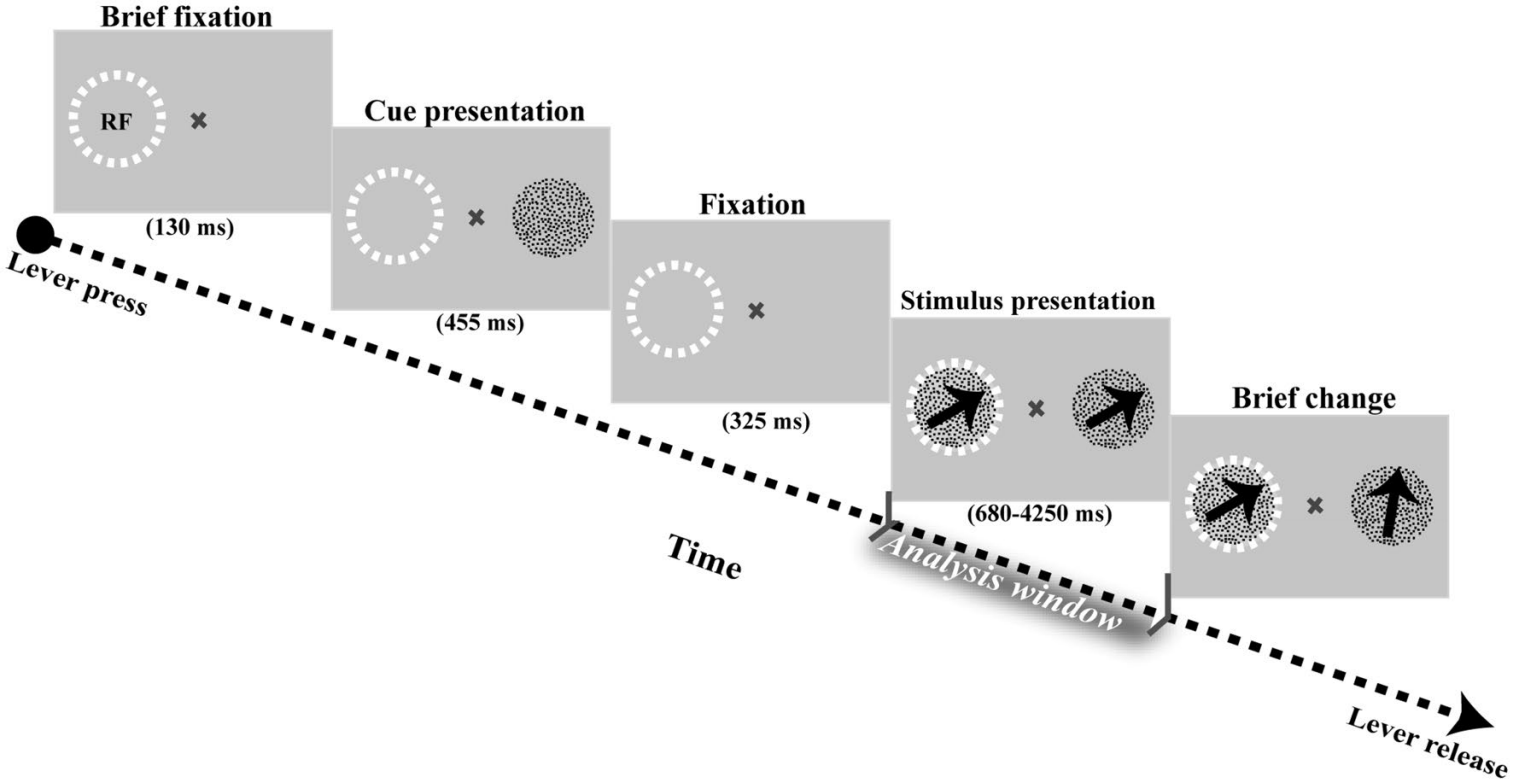
Experimental procedures. The monkey began each trial of the task by pressing a lever bar and maintaining its gaze on a fixation point (multiplication sign) for 130 ms. Then a static RDP appeared for 455 ms to show the position of the target stimulus on the screen to the animal. After the cue presentation, the screen became blank for 325 ms, and the monkey was required to preserve its gaze on the fixation point then. Next, two moving RDPs with the same direction of motion emerged on screen for a random period of 680–4250 ms. Immediately after, a direction change occurred in one of the two RDPs and the monkey was instructed to release the lever if the change happened in the cued position. The dashed circle indicates the classical RF of the neuron on the screen. The circle was not depicted to the monkey in the actual task experiments. All analyses were carried out on the analysis window during the stimulus presentation period, delineated by a grey shadow in the figure.

We trained a male macaque monkey to attend to one of two stimuli presented on the monitor screen. The animal was seated in a custom-built primate chair, fixing its head and stabilizing its body from excessive movements while performing the task. Each trial was started by touching a lever bar and maintaining the gaze on a central fixation point for 130 ms. Upon gazing at the central fixation, a static random dot pattern (RDP) appeared for 455 ms, presenting the position of the target stimulus on the screen. Following a blank period (325 ms), two moving RDPs appeared inside and outside the receptive field (RF) for a random duration of 680 ms to 4250 ms. The two RDPs had the same size, adjusted manually to be matched the size of the classical RF of the neuron recorded in each session. They were always positioned symmetrically around the fixation point. The direction of motion in both target and non-target (distractor) RDPs were the same, selected randomly from eight possible directions (between 0–360° with steps of 45°) in the polar coordinate system. After passing a random period from displaying the RDPs, a brief direction change occurred in one of two RDPs. The monkey was rewarded with a drop of juice after correctly identifying the change in the target stimulus and ignoring the change in the distractor. The reward was not delivered when the monkey (i) released the lever for a distractor change, (ii) broke his gaze on the fixation spot during the trial, or (iii) responded too late after the target change. Overall, the monkey could successfully identify the changes in the target stimulus for 86% of the trials without fixation breaks. He imperfectly finished 3 and 11% of trials either by replying to non-target changes (false alarms) or by terminating them without executing any response (miss trials), respectively. The task design and behavioral paradigm are illustrated in Fig. 1.

We recorded the extracellular activity of MT neurons using a multi-channel recording system (Mini-Matrix, Thomas Recording, and Plexon data acquisition system, Plexon Inc.). The extracellular signals were split into local field potentials (LFPs; 1–300 Hz) and single-unit neural activities (SUAs) using 4-pole Bessel low-pass and high-pass digital filters at cut-off frequencies of 300 Hz and 500 Hz, respectively. The filters were hardware embedded in the recording system (Plexon, Dallas, TX, USA), set as the default option to extract extracellular signal components. Then, the SUAs and the LFPs were amplified and digitized at 40 and 1 kHz, respectively. We eliminated the power line noise (50 Hz) from the LFPs using a non-causal 4th-order Butterworth notch filter. The SUA was isolated online using Plexon’s online sorter application system. Overall, the collected data were recorded from 111 cortical sites using up to five parallel electrodes, advanced into brain tissue to isolate direction-selective MT neurons overlapping RFs (linear electrode arrangement with an inter-electrode distance of 300 µm). These electrodes were not chronically implanted but were inserted simultaneously in each experimental session. We identified MT sites using their anatomical location in the cortex (by employing structural MRI imaging) and their physiological properties, which were direction-selective when the average diameter of their RF was almost equal to the RF eccentricity. It is clear that the RF centers of MT neurons for different locations are anatomically located along the superior temporal sulcus in the cortex.

### Data-analysis procedure

Details of quantitative procedures and analytical approaches applied to recordings are described in the following sections. All our analyses of the recorded data were implemented by taking advantage of MATLAB software (R2017b; MathWorks, Natick, MA, United States).

### Trial selection approach

Trials whose target change was correctly reported by the monkey (hit trial) were considered for further analyses. These trials were divided into two subclasses of “target-in” or “target-out” according to whether the target RDP appeared inside or outside the RF, respectively. We equalized the number of trials between the two subclass; therefore, there were 4661 trials in each subset. We further categorized the hit trials into “fast” and “slow” subsets based on the animal’s reaction time (RT). For this purpose, trials recorded from each neuron were sorted depending on their RTs in an ascendant order and subdivided into five equal subsets^60^. Then, trials belonging to the first and the last subset were labeled as the fast and slow trials, respectively. This segregation routine was frequently used in other studies^26,60–65^ investigating the relationship between attention\working memory-dependent modulation in the early visual cortex and behavioral improvement. We did not analyze the trials in the middle subsets due to no substantial differences in their neural correlates of behavior (reference^65^, Supplementary materials, Fig. 5). All analyses of our study were carried out on SUAs and LFPs, recorded from the same electrode during the stimulus presentation period. The analysis window bore 1500 ms length, starting from about 600 ms following the stimulus onset and ending before stimulus change. We ensured that this analysis window was far from the stimulus onset, which produces stimulus-induced changes in the brain’s rhythmicity (oscillatory activity)^66–69^. The supplementary figure S2 (Monkey H) in reference^6^ illustrated the temporal dynamic of the stimulus-evoked responses induced by the onset of motion stimuli.

### Data analysis

#### Instantaneous frequency

**Figure 2:**
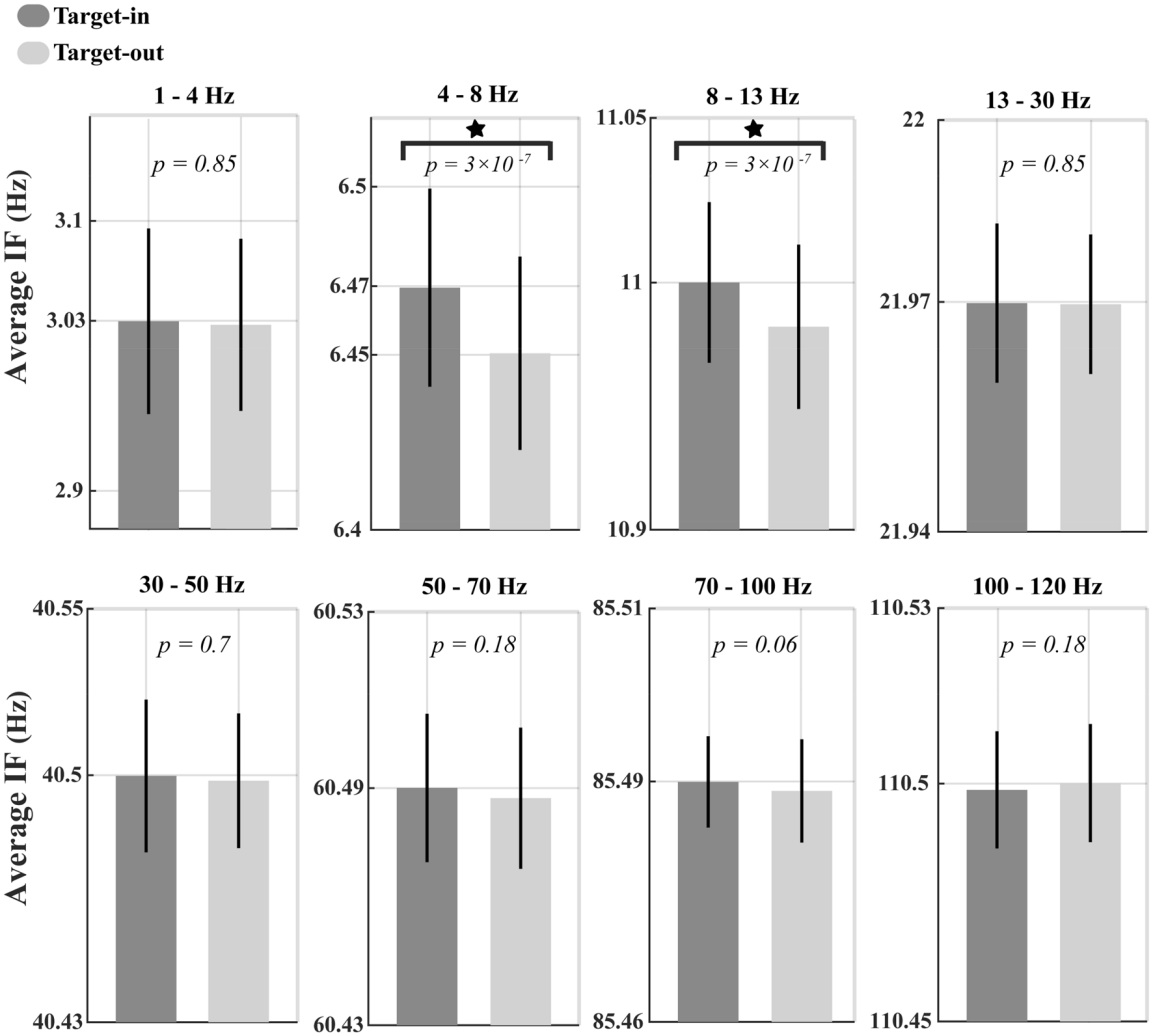
Frequency specificity of MT neurons in processing goal-oriented stimuli. The light and dark bars represent the average instantaneous frequency of neurons in Delta (1–4 Hz), Theta (4–8 Hz), Alpha (8–13 Hz), Beta (13–30 Hz), low-Gamma (30–50 Hz), mid-Gamma (50–70) and high-Gamma (70–120) bands at the target-in and the target-out conditions, respectively. Error bars represent the ± 1 STD. The inset *p*-values depict the significance level of differences between the target-in and the target-out conditions for each frequency band (two-sided sign-test). Asterisks indicate frequency bands with a significant difference in the average instantaneous frequency of neurons between the two target conditions (*p* < 10^−6^, two-sided sign-test).

**Figure 3:**
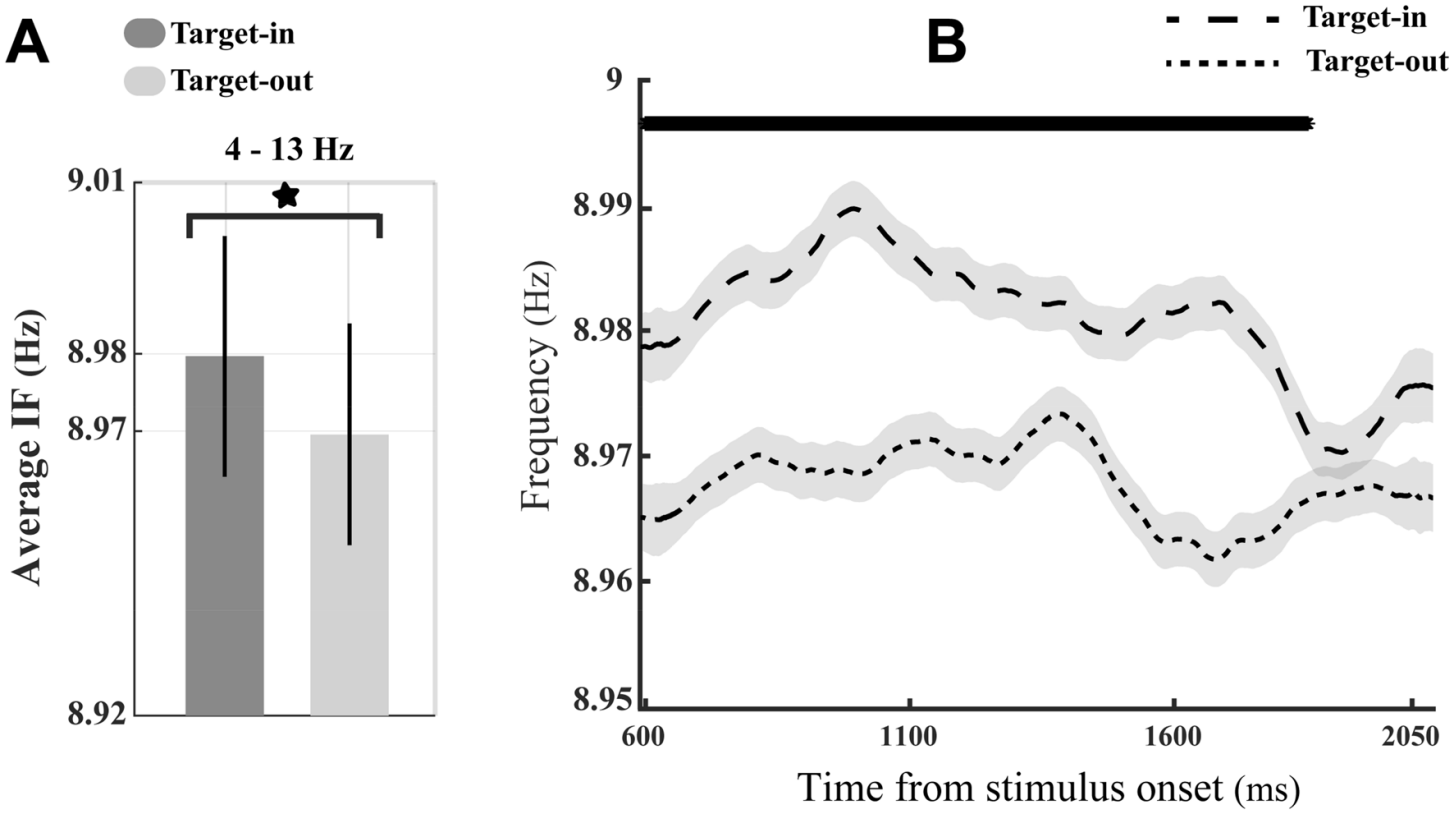
Modulation of the theta–alpha IF in trials with a resembling SNR. (**A**) The average IF of neurons at the target-in (the dark bar) and the target-out (the light bar) conditions. Despite estimating the IF in the frequency range of 4–13 Hz, the Y-axis was manually adjusted to provide the best illustration of the significant difference between light and dark bars. The asterisk demonstrates a significant difference in the average IF between the two task conditions (*p* < 1.3 × 10^−7^, two-sided sign-test). Error bars show the ± 1 STD. (**B**) The temporal variation of the theta–alpha frequency during the stimulus presentation period. The thick-black line indicates a time window with a significant discrepancy in the IF of neurons between the two task conditions (*p* < 0.008, two-sided Wilcoxon rank-sum test with FDR correction for multiple comparisons).

We calculated the time-varying estimate of the instantaneous frequency (IF) using a modified form of a method developed by M.X Cohen^22^. First, single-trial LFP signals were band-pass filtered using a zero-phase, windowed harmonic wavelet transform (WHWT) filter^59^. The following equation expresses the WHWT in the frequency domain;

1 $$W_{H} (f) = \left\{ {\begin{array}{*{20}l} {A + B\cos \left( {2\pi \left( {sf - \eta } \right)} \right)} \hfill & {2\pi \left| {\overline{f} - f} \right| < \pi {/}s} \hfill \\ 0 \hfill & {{\text{elsewhere}}} \hfill \\ \end{array} } \right.$$

where $\overline{f} = \eta /s$ represents the frequency center, $\eta$ shows the frequency center of the harmonic wavelet at scale $s = 1$ (i.e. the mother wavelet), $b_{f} = 1/s$ defines the bandwidth of wavelet function and $A,B$ are two constant parameters adjusted on $A = B = 1/\sqrt 3$. We filtered LFPs in a frequency range between 1 to 250 Hz, with a step of 1 Hz and a bandwidth of 4 Hz. Since the WHWT filter has a Gaussian shape in the frequency domain, many frequency-overlapping filters (using 250 numbers of WHWTs) were used to avoid an impending bias of estimated IF toward the peak frequency of the Gaussian. The filtered signals obtained by applying WHTW on an LFP comprised a real ($x\left( t \right)$) and an imaginary ($y\left( t \right)$) part. We next used $x\left( t \right)$ and $y\left( t \right)$ to calculate the IF for each filtered signal by employing a two-point finite impulse response (FIR) differentiator, defined as follows^70^;

2 $$F(t) = \frac{1}{2\pi T}\arctan \left( {\frac{{x(t)y\left( {t + T} \right) - x(t + T)y(t)}}{{x(t)x\left( {t + T} \right) - y(t)y(t + T)}}} \right)$$

where $T = 1/f_{s}$ and $f_{s}$ determine the sample period and sampling frequency of the LFP signal (1 kHz), respectively. In contrast to traditional approaches to IF calculation, the two-point FIR differentiator estimates the IF with a minimum number of non-physiological frequency jumps arising from temporal decreases in the signal-to-noise ratio of the filtered signal^70^. The estimated IF was further filtered ten times using median filters with ten different window widths equally spaced between 10 and 400 ms^22^. The median of different median-filter windows estimated the IF in each trial. Applying multiple median filters can prevent spectral leakage of spike waveform^71,72^ into IF estimation. Next, IFs of each trial (with the resolution frequency of 1 Hz) were averaged over each frequency range, including Delta (1–4 Hz), Theta (4–8 Hz), Alpha (8–13 Hz), Beta (13–30 Hz), low-Gamma (30–50 Hz), mid-Gamma (50–70) and high-Gamma (70–120). Now, we measured the average IF in seven different frequency bands for each trial of a recording site. Finally, the IFs estimated on each frequency band were averaged across trials of each recording site. Figures 2, 3A, 5A, 6A and 7A represents the IF averaged over the analysis window and recording sites in a frequency band.

#### Correlation between IF modulation and modulation in neural firing-rates

**Figure 4:**
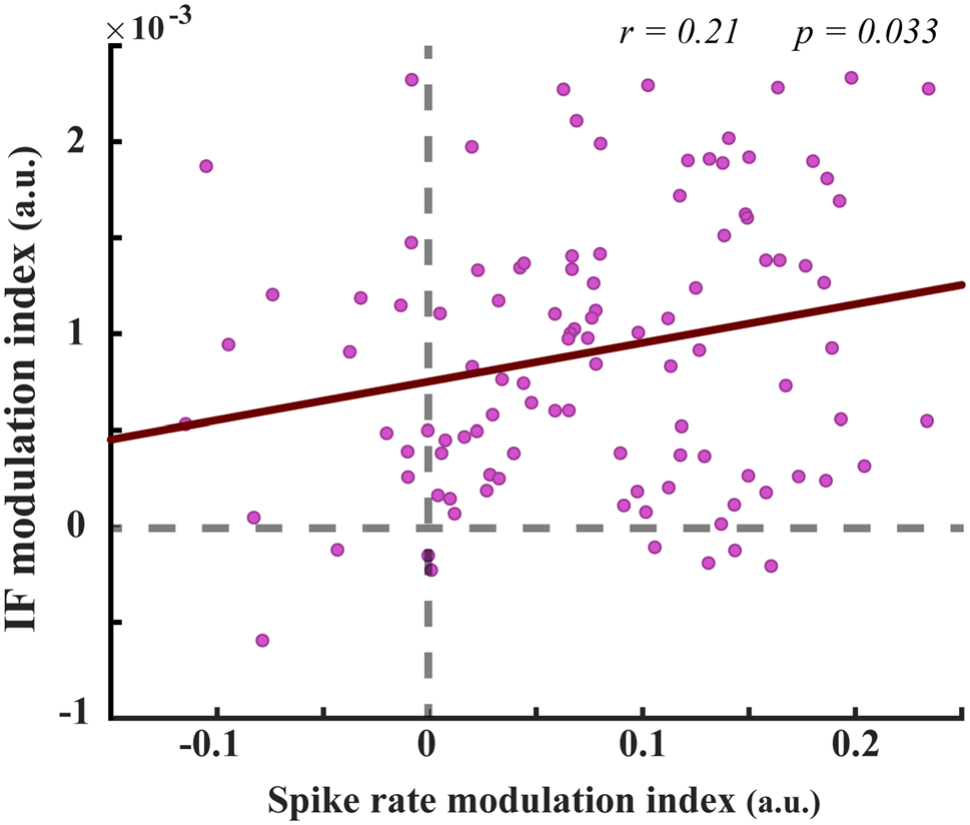
Correlation between neural firing rate variations and variations in theta–alpha IFs of input oscillatory activity. Individual neurons are each represented by a filled circle in the graph. Inset numbers reveal the correlation strength (r, using the Spearman correlation method) and the degree of its significance (*p*) for the shown dataset.

**Figure 5:**
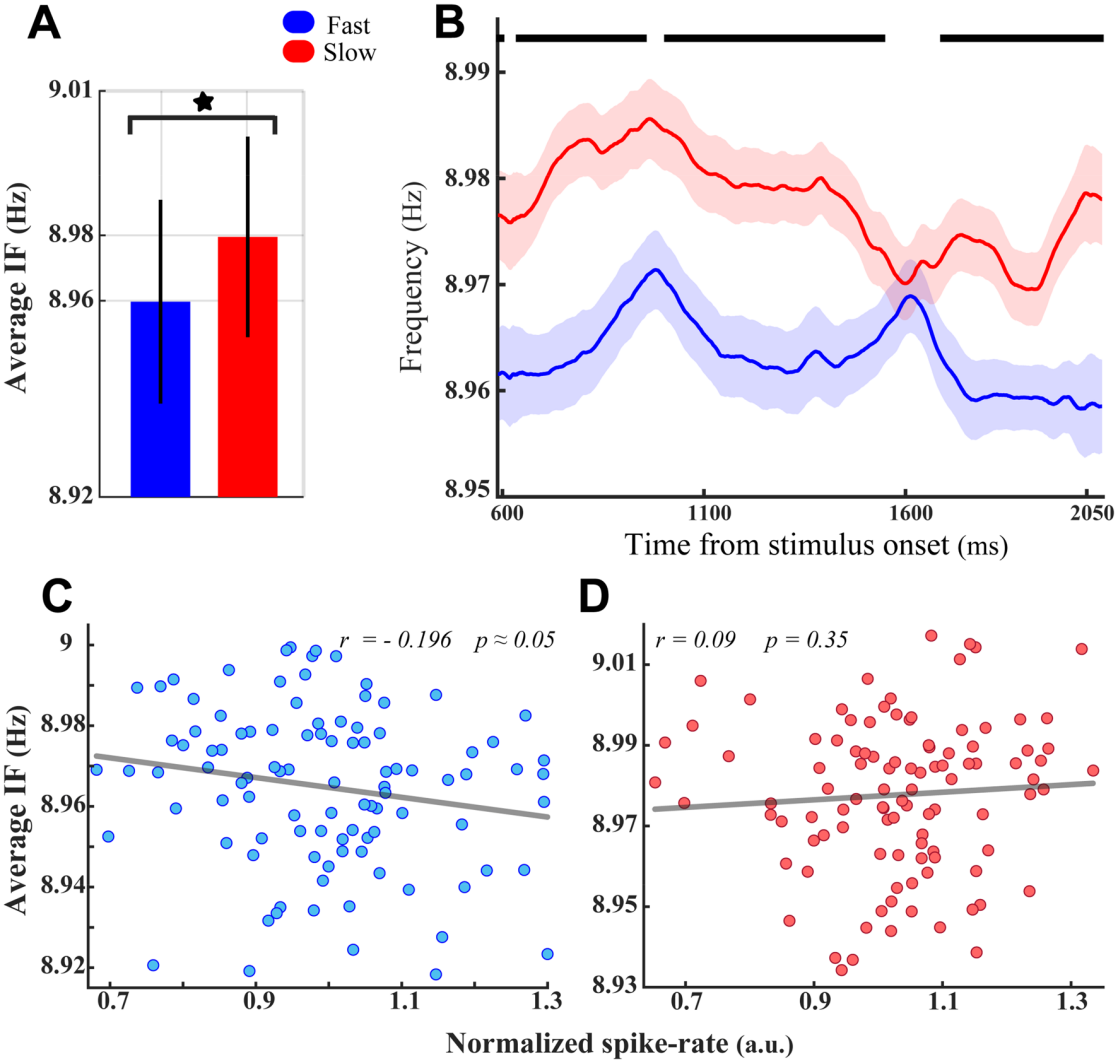
Functional significance of frequency modulation in the theta–alpha band; the effect of frequency modulation on behavioral RTs (**A**) Average theta–alpha frequency of neurons in fast RT trials versus slow RT trials. Despite estimating the IF in the frequency range of 4–13 Hz, the Y-axis was manually adjusted to provide the best illustration of the significant difference between light and dark bars. The asterisk depicts a significant difference in the average IF between the two categories (*p* < 7 × 10^−5^, two-sided sign-test). Error bars show the ± 1 STD. (**B**) The temporal dynamics of average IFs, shown in panel A, for the fast versus slow RT trials. Thick-black lines indicate time windows with a significant difference between RT conditions (*p* < 0.042, two-sided Wilcoxon rank-sum test with FDR correction for multiple comparisons). Error bars represent the SEM. (**C**,**D**) The spike rate of each neuron plotted against the average theta–alpha IF of the LFP in fast RT (**C**) and slow RT (**D**) trials. Inset numbers in panels (**C**,**D**) represent the correlation magnitude (*r*, using the Spearman correlation method) and the degree of its significance (*p*).

**Figure 6:**
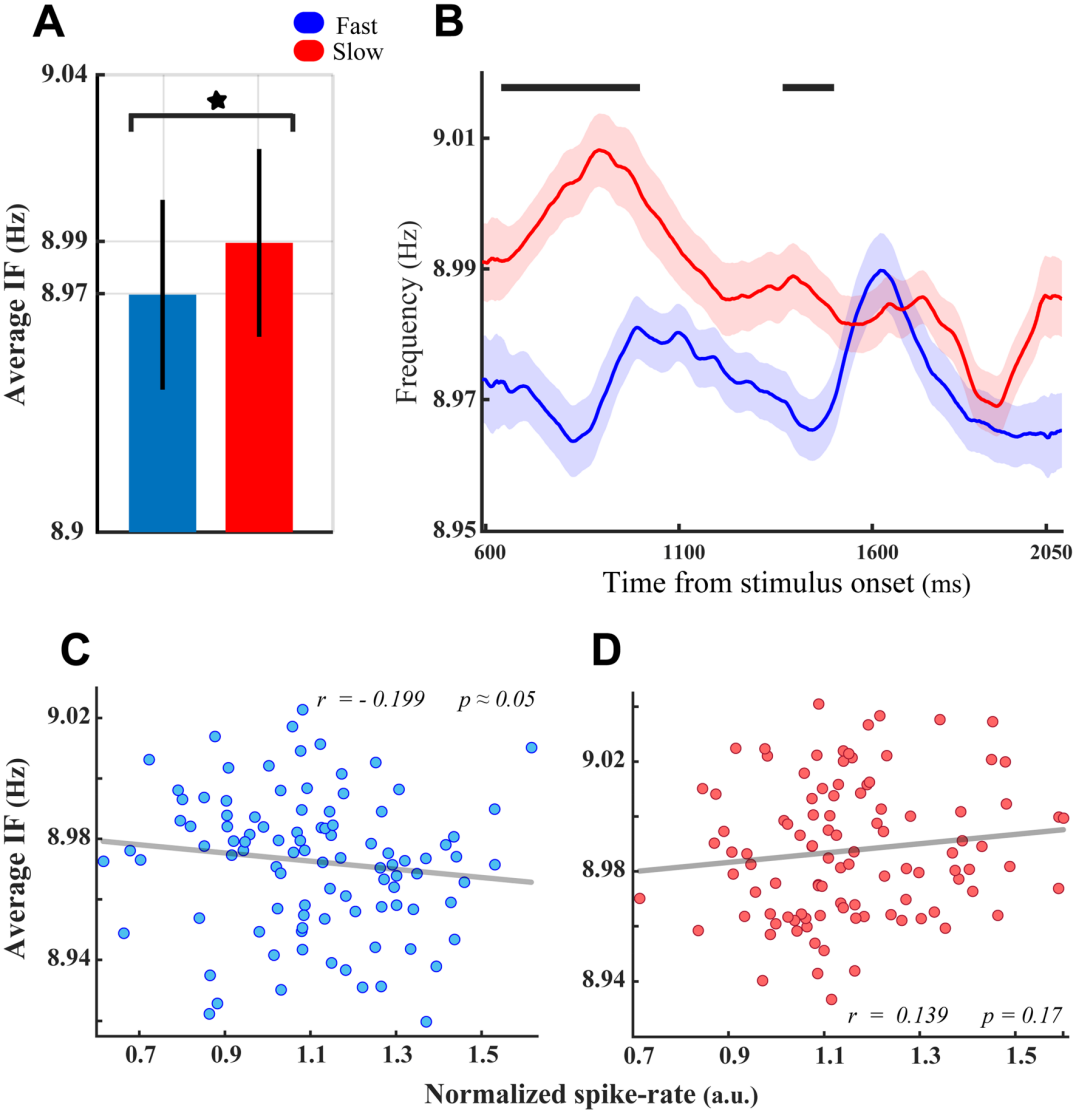
Functional significance of the theta–alpha frequency modulation at the target-in condition. The detailed description of (**A**–**D**) is the same as the panels shown in Fig. 5. (**A**–**D**), respectively, except the significance levels in (**A**) (*p* < 8 × 10^−5^, two-sided sign-test) and (**B**) (*p* < 0.014, two-sided Wilcoxon rank-sum test with FDR correction for multiple comparisons). Likewise, the inset numbers in (**C**,**D**) display the results of correlation analyses in fast and slow RT trials, respectively.

We measured the magnitude of changes in the firing rate of SUAs against the magnitude of changes in the IF averages for every recording site using target-in and target-out trials. The two parameters were computed on each site using SUAs and LFPs recorded from the same electrode. For this purpose, the spike-rate modulation index (SP_MI_) and the IF modulation index (IF_MI_) was computed using the following equations;

3 $${\text{SP}}_{{{\text{MI}}}} = \frac{{Sp_{in} - Sp_{out} }}{{Sp_{in} + Sp_{out} }}$$

4 $${\text{IF}}_{{{\text{MI}}}} = \frac{{IFav_{in} - IFav_{out} }}{{IFav_{in} + Fav_{out} }}$$

where $Sp$ and $IFav$ represent the trials-averaged firing rate of an SUA and the trials-averaged of IF, respectively, both measured from the same recording site and on the same analysis window. Furthermore, $\left( \cdot \right)_{in}$ and $\left( \cdot \right)_{out}$ implicate the parameters estimated at the target-in and target-out conditions, respectively. We pooled pairs of (SP_MI_, IF_MI_) obtained for individual MT sites and then removed those outlier pairs that exceeded the mean ± 2 × standard deviations (SD) of the pooled data (6 outlier pairs removed from the total 111 ones). Finally, the Spearman correlation was employed to measure the magnitude of the correlation between SP_MI_ and IF_MI_ values (see Fig. 4). It is worth noting that we conducted the same routine to eliminate outliers from analyses of correlation shown in Figs. 5C,D, 6C,D and 9B,C.

#### Spike-frequency Granger causality for fast and slow trials

Having segregated the fast and slow trials using the method described above, we selected those trials from each MT site whose spike rates (SR) satisfied the following thresholds;

5 $$Tr_{1} \le SR \le TR_{2}$$

where $Tr_{1} = 9$ and $Tr_{2} = 84$ are two thresholds calculated as follows;

6 $$Tr_{1} = median(SR_{all} ) - mad(SR_{all} )$$

7 $$Tr_{2} = median(SR_{all} ) + 2 \times mad(SR_{all} )$$

where $SR_{all}$ is a vector comprised of spike rates from all MT sites, $median\left( \cdot \right)$ and $mad\left( \cdot \right)$ are two operators computing “median” and “median absolute deviation”, respectively. We next measured the causality relationship between the IF and spiking activity of selected trials in each MT site using a method proposed by Gong et al.^73^. Indeed, the SR thresholds (defined in Eq. (5)) made the causality estimates of different recording sites comparable due to eliminating trials with outlier SRs from each recording site. It is important to note that most recording sites (98 sites out of 111 ones) were preserved after applying the SR thresholds. Since the causality approach was conducted on each recording site for fast RT trials (Fig. 8), the removed recording sites might include no fast RT trials passing the SR threshold criteria.

#### Spike-triggered average of IF (STA_IF_) and spike-frequency directionality measure

We calculated STA_IF_ to examine IF variations around MT’s spikes in the frequency range of 4–13 Hz. For this purpose, segments of the IF in a 350 ms time window surrounding spikes of each trial were extracted and then averaged across trials (Fig. 8). All (fast RT) trials containing at least one spike were used for this analysis. Furthermore, we statistically compared the IF variation between the time window preceding the spike and the time window after it^74,75^. For this purpose, we calculated the differences between individual IFs of the time windows and the IF value at the spike event. We then subtracted the absolutes of IF differences in the second half window (i.e. the 350 ms window after the spike event) from the corresponding absolutes of IF differences in the first half window (i.e. the 350 ms window before the spike event), both of which have an equal time interval to the spike time. Finally, the resulting values of subtraction averaged across all samples of a segment. By applying the same analytical procedure for whole segments extracted, we obtained a distribution of IF variations whose median sign represents directional influences between MT’s firing activity and MT’s IF input^74,75^. With this, our results revealed a significant positive median, implicating the IF input to the MT neuron causes spikes to generate ($p < 0.04,$ Wilcoxon signed-rank test).

#### Correlation between spike-phase coupling (SPC) and the neural IF

SPC represents oscillatory phase synchrony between the LFP and the single-unit spiking activity, recorded simultaneously from a single electrode. To estimate the SPC in the fast and the slow RT trials of the target-in condition, we employed the WHWT to filter the LFPs in a high-gamma frequency range of 190 to 210 Hz, with a step of 2 Hz and a bandwidth of 2 Hz. The frequency range of 190–210 Hz was chosen based on a recent study, indicating the maximum SPC differences in that frequency band between the fast and the slow RT trials^6^. Next, the instantaneous phase (IP) of filtered signals was calculated by taking the tangent inverse of the ratio of its imaginary part to the real part. Finally, we estimated the SPC on each trial by computing the magnitude of the circular average of IPs simultaneous to the spikes^6^. The SPC methods are susceptible to the number of spikes in the analysis window^76–78^. To eliminate any potential bias to the spike rate, we used a thinning procedure^78,79^ to select trials whose number of spikes was higher than the median of spike rates in each recording site. With this, 101 out of all 111 recording sites remained. Furthermore, we randomly removed surplus spikes from each remaining trial until the median spike rate was reached. Having applied the same procedure on trials of a given MT site for 1000 repetitions and averaged the resulting SPCs, we obtained a reliable SPC in each frequency band. The SPC of individual trials had also been smoothed with a window of 6 Hz before computing its average across frequency bands. Eventually, having computed the average SPC and the average IF in each recording site, we measured the correlation between these two quantities across different MT recording sites using the Spearman method. Recording sites showing either the average SPC or the average IF exceeded the Mean ± 1 × SD of the pooled SPCs or mean ± 2 × SD of the pooled IFs was eliminated from the correlation analysis.

## Results

To determine how the instantaneous oscillation frequency of visual neurons varies subject to task demands, we recorded single-unit activities and local field potentials (LFP) from the area MT of a macaque monkey performing a change detection task. The monkey was trained to covertly attend to one of two coherently random dot patterns (RDP) to detect a brief alteration in its direction of motion (Fig. 1, see “Methods”). The monkey’s performance correctly reporting the changes in the target RDPs was 86% (percentage of hit trials) without breaking its eye fixation. We used LFP recordings of hit trials along the stimulus presentation period to measure the average instantaneous frequency (IF) of MT’s population activity at the different frequency bands, including delta (1–4 Hz), theta (4–8 Hz), alpha (8–13 Hz), beta (13–30 Hz), low-Gamma (30–50 Hz), mid-Gamma (50–70) and high-Gamma (70–120) (Fig. 2). Comparing the average IFs between the target-in and the target-out experiments revealed a significant upward shift in the IF of the theta–alpha band (4–13 Hz) among the recorded population of MT neurons at the target-in condition (*p* < 10^−6^, two-sided sign-test). However, analyses of the theta-alpha IF of error trials did not show a significant difference in the average theta-alpha IF between the target-in and target-out conditions. (*p* = 0.073, two-sided sign-test, Supplementary materials, Figure S1). The IF modulation is not due to potential differences in reward volumes delivered to the monkey; it was the same across the hit trials. Future research using a behavioral task with variable reward-delivery contingency could reveal whether the reward volume can modulate the IF in the brain. To rule out any confound due to differences in the signal-to-noise ratio (SNR) of LFPs that may explain such significant differences in the theta-alpha IF, we selected trials of each recording site bearing no significant differences (*p* > 0.99, two-sided Wilcoxon rank-sum test) in theta-alpha power between the target-in and the target-out conditions. Of this selection, the difference in the average IFs remained significant among two experimental task conditions (*p* < 1.3 × 10^−7^, two-sided sign-test, Fig. 3A), suggesting that modulation of the theta-alpha IF among the population of MT neurons does not associate with the changes in the SNR of their activities. Conceptually, an IF modulation as high as 0.02 Hz (shown in Fig. 3A) likely means that theta-alpha oscillation cycles in the target-in condition are shorter by about 20 ms than those in the target-out condition. We also observed a significant frequency modulation in the theta-alpha band from the beginning of the analysis window, which lasted before the end of it (*p* < 0.008, two-sided Wilcoxon rank-sum test with FDR correction for multiple comparisons, Fig. 3B). These results suggest that modulation of theta-alpha IF at the population level of MT neurons is spatially selective for the stimulus positioned at the inside RFs of neurons (target-in versus target-out conditions).

In order to determine whether changes in theta-alpha IF, as variations in the frequency of oscillation input^22^ to MT neurons, reflect changes in their spike outputs, we calculated the correlation between spike-rate alternations (as a measure of changes in neuronal excitability^80,81^) and IF modulations. For this purpose, we used trials of the target-in and the target-out conditions recorded at each site (see “Methods”). The results clearly showed that the variations in theta-alpha IFs were significantly accompanied by changes in MTs’ spike rates (*r* = 0.21*, p* = 0.033, Spearman correlation, Fig. 4). In other words, the significant positive correlation between the two quantities mentioned above indicates that fluctuations of the theta-alpha IFs are directly reflected in the fluctuations of firing rates of individual MT neurons. Despite this result, neuronal spiking activity did not show a significant correlation with the average theta-alpha IF neither at the target-in (*r* = 0.17*, p* = 0.089, Spearman correlation, Figure S2.A) nor at target-out (*r* =  − 0.099*, p* = 0.31, Spearman correlation, Figure S2.B) conditions. Assuming the analyzed neurons were recorded from a local network in area MT, Figure S2 might reflect that the relation between the frequency input and the spiking output of the network neurons is not spatially selective to the target stimulus location. However, most of the single neurons (83 out of 105, which have positive x–y values in Fig. 4) depicted selective enhancement in their spiking activity and input frequency by attending to the target-in condition.

To assess the functional significance of theta-alpha IF modulation^26^, we examined its impact on behavioral performance. Since the monkey correctly finished the task over 85% of trials, there were not enough error trials to conduct a convincing accuracy-related analysis. We instead advanced our analysis using behavioral reaction time (RT) and divided trials accordingly into fast RT and slow RT groups, regardless of their target locations\ conditions (see “Methods”). It is evident in Fig. 5A that the slow RT trials were associated with a significantly higher average alpha-theta IF than the fast RT trials (*p* < 7 × 10^−5^, two-sided sign-test), of particular across most moments of the analysis window (*p* < 0.042, two-sided Wilcoxon rank-sum test with FDR correction for multiple comparisons, Fig. 5B). Despite this significant enhancement, which likely improves the signal-to-noise ratio of frequency input to neurons in slow RT trials, analyses of the correlation between the theta–alpha IF and neural spike rate illustrates a marginally significant negative correlation in fast RT trials (*r* =  − 0.196, *p* ≈ 0.05, Spearman correlation, Fig. 5C) but no significant correlation in the slow RT trials (*r* = 0.09*, p* = 0.35, Spearman correlation, Fig. 5D). It is worth noting that such modulation of theta–alpha IFs is not simply an artifact of the difference in the oscillatory theta–alpha powers of LFPs between the fast and the slow RT trials (*p* > 0.08, two-sided sign-test). Remarkably, the comparison of Fig. 5C with Figure S2 indicates contrasting outcomes for the correlation between the theta–alpha IF and spiking activities of MT neurons. These contrary results might imply that the theta–alpha IF, as frequency input oscillations projecting top-down influences to the MT area^22,26^, can regulate the firing activity of MT neurons at the levels of the single-unit (Fig. 4) and the local network (Fig. 5C) in distinct fashions. Moreover, these opposite observations might systematically mediate the routing of the relevant information from the visual cortex into higher-order associated areas efficiently (*see also Discussion for more details*).

**Figure 7:**
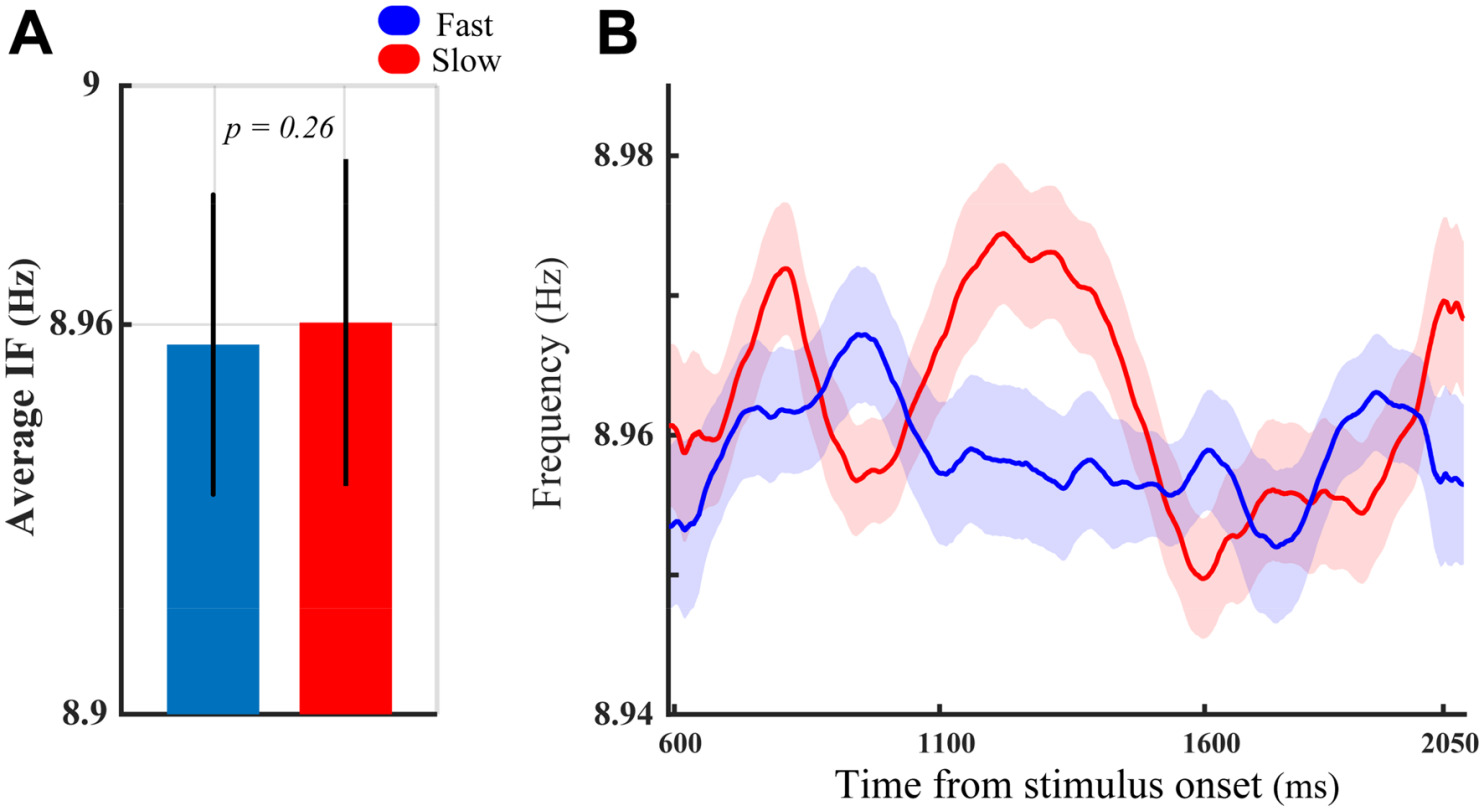
Functional significance of the theta–alpha frequency modulation at the target-out condition. The detailed explanation of (**A**,**B**) is the same as the panels shown in Fig. 5. (**A**,**B**), respectively, except the significance levels in (**A**) (*p* > 0.25, two-sided sign-test) and (**B**) (*p* > 0.05, two-sided Wilcoxon rank-sum test with FDR correction for multiple comparisons).

The functional role of alpha–theta IF modulation was further explored by examining the impact of the target stimulus locations (experimental task conditions) on the results obtained in Fig. 5. We first conducted the same analyses of behavioral performance for the trials recorded at the target-in condition. These analyses illustrated a significant theta–alpha IF enhancement in slow RT trials compared with the fast RT trials (Fig. 6A; *p* < 8 × 10^−5^, two-sided sign-test, Fig. 6B; *p* < 0.014, two-sided Wilcoxon rank-sum test with FDR correction for multiple comparisons), resembling those displayed in Fig. 5A,B. However, Fig. 6B depicts a different IF modulation dynamic than the one in Fig. 5B, in which the significant IF differences emerged at the initial moments of the analysis window and progressively disappeared as it got closer to the stimulus change time at the end of it. The correlation between the average theta–alpha IF and neural spike rates across different recording sites (depicted in Fig. 6C,D) showed congruent results with those depicted in Fig. 5C,D. Accordingly, despite the firing rate of MT neurons being significantly suppressed by increases in the magnitude of the theta–alpha IF in fast RT trials (*r* =  − 0.199*, p* ≈ 0.05, Spearman correlation, Fig. 6C), there is no significant correlation between neural spike rate and theta–alpha IFs in slow RT trials (*r* = *0.139, p* = 0.17, Spearman correlation, Fig. 6D). Repeating the analysis of the behavioral performance for the trials of the target-out condition did not show any significant differences in the average theta–alpha IF between the fast and the slow RT trials (Fig. 7A; *p* > 0.25, two-sided sign-test, Fig. 7B; *p* > 0.05, two-sided Wilcoxon rank-sum test with FDR correction for multiple comparisons).

**Figure 8:**
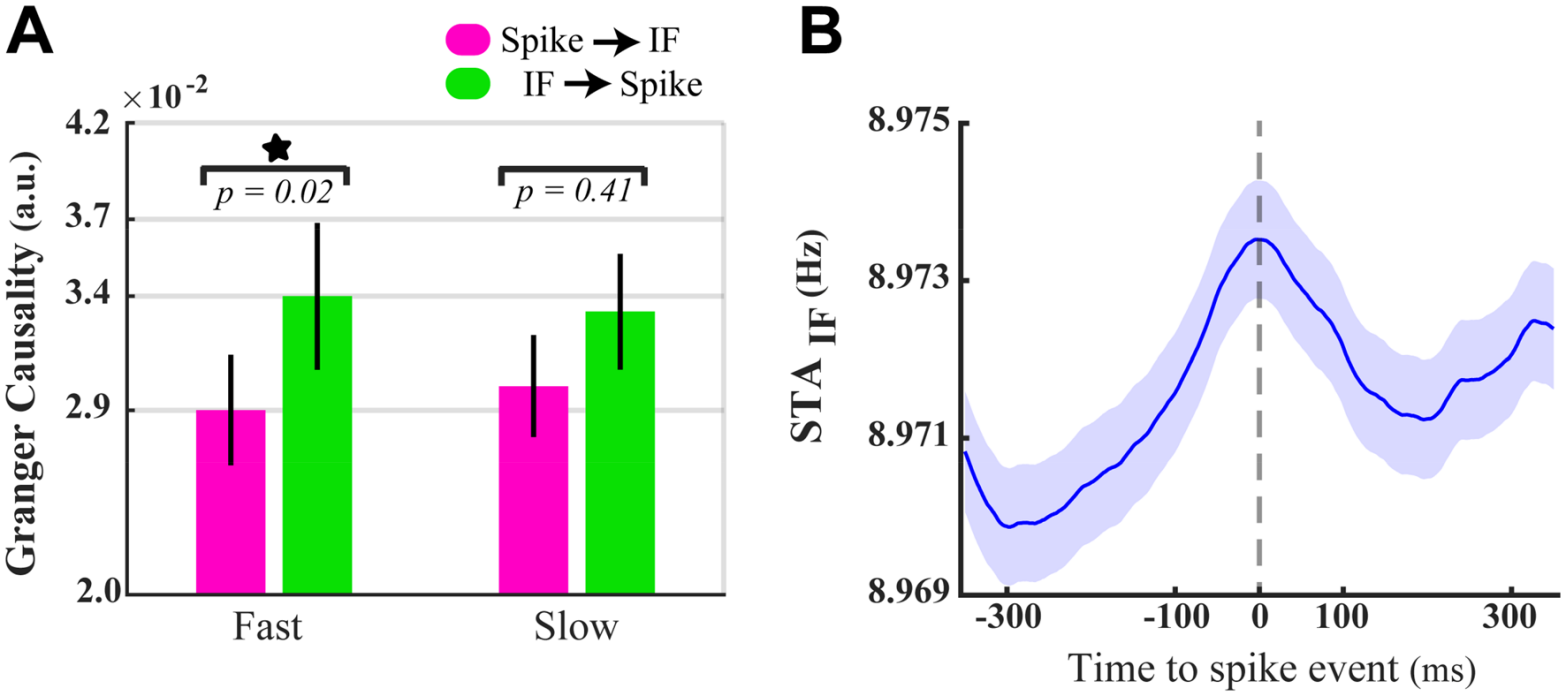
Granger causal influence between spike output and frequency of input oscillatory activity. (**A**) Directional effect of the theta–alpha IF on spiking activity of neurons (green color) and vice versa (purple color) among fast and slow RT trials. The Asterisk indicates a significant difference between two opposite causal influences in fast RT trials (*p* < 0.03, Wilcoxon signed-rank test). The error bars show ± 1 STD. (**B**) Spike triggered averages of IF in the theta–alpha band. The mid-dashed line illustrates the time when neurons were discharging. The error bars represent the ± SEM.

We next examined the directional influences of theta–alpha IFs on single neurons’ spike rate in the fast and the slow RT trials (see “Methods”). The result of Fig. 8A confirmed that the input theta–alpha IFs has a significant causal influence on the spike output of single neurons only in fast (*p* < 0.03, Wilcoxon signed-rank test) rather than slow RT trials (*p* > 0.4, Wilcoxon signed-rank test). One potential concern is that the measure conducted to examine causality sometimes illustrates bias or high variance estimates, leading to spurious results in neuroscience studies^82^. Therefore, to further investigate IF-induced changes in the spiking activity of single neurons, we computed the spike-triggered average of IF (STA_IF_) in fast RT trials (see “Methods”). This analysis allowed us to measure fluctuations of the theta–alpha IF around the moments at which action potentials (spikes) happened. We found that the magnitude of the IF variation in a 350 ms window preceding the spikes is significantly larger than the one following it (*p* < 0.04, Wilcoxon signed-rank test, see “Methods”). This observation also confirmed that input oscillation frequency at the theta–alpha band has a directional impact on single neurons’ discharging^74,75^.

**Figure 9:**
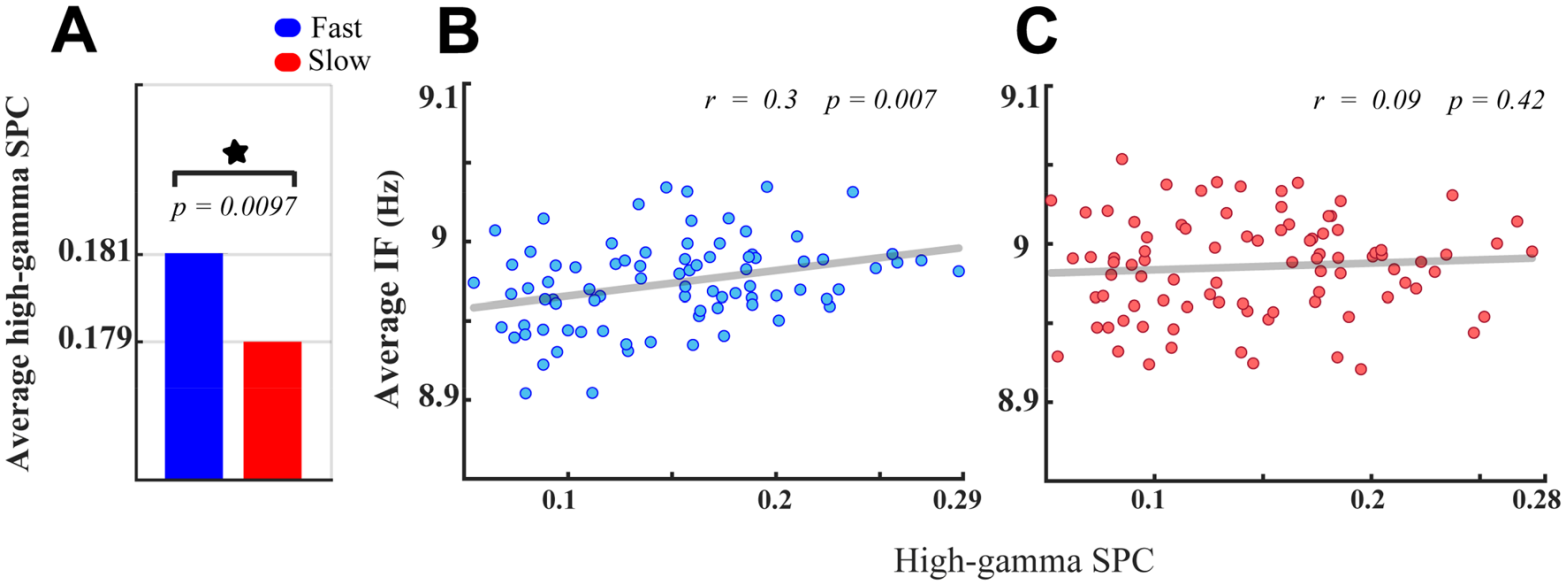
Correlation of neural synchronization in high-gamma (190–210 Hz) with the average theta–alpha IF. (**A**) High-gamma SPC averages of neurons in fast and slow RT trials. The asterisk demonstrates a significant SPC difference in the two types of trials (*p* < 0.01, two-sided sign-test). (**B**,**C**) The average theta–alpha IF (Y-axis) plotted against the high-gamma SPC (X-axis) at individual recording sites (represented by a blue \ red filled circle) using its fast (**C**) and slow (**D**) RT trials. The inset numbers in (**B**,**C**) reveal the result of conducting a Spearman correlation test on X–Y variables.

We then assessed whether higher instantaneous theta–alpha frequency results in enhanced synchronization selectively among neurons processing the target stimuli (i.e. the target-in condition). Our previous study illustrated that the strength of neural synchronizations in the high-gamma frequency band (190–210 Hz) is modulated depending on the speed of the behavioral action^6^. To test the suggested hypothesis, we first conducted spike-phase coupling (SPC, see “Methods”) to measure the magnitude of neural synchrony in the high-gamma frequency band in the fast and the slow RT trials. The analysis of SPCs revealed that the magnitudes of the high-gamma SPC significantly increased in the fast compared with the slow RT trials at the population level of MT neurons (Fig. 9A; *p* < 0.01, two-sided sign-test). We next assessed the SPC correlates of the average theta–alpha IF in fast and slow RT trials. The result demonstrated positive correlations between SPCs and the theta–alpha IFs, which are significant only in the fast (*r* = *0.3, p* = 0.007, Spearman correlation, Fig. 9B), but not in the slow RT trials (*r* = *0.09, p* = 0.42, Spearman correlation, Fig. 9C). Given that frequency modulations of the theta–alpha oscillation modulate neurons’ spiking outputs (see Fig. 8), the theta–alpha IF as a neurophysiological regulatory factor can control the synchronization strength among MT neurons (^56^, *see also Dissuasion for more details*).

## Discussion

In the present study, we contrasted the oscillatory activity of MT neurons during two conditions of a change detection task. We found that the frequency of neural oscillations in the theta–alpha band differs between these experimental conditions. The frequency increased among MT neurons when the monkey correctly detected stimulus changes in the cued location inside the neurons' RF compared with those outside the neurons' RF. (Figs. 2, 3). Moreover, our results illustrated that the firing rate of individual MT neurons is modulated along with the frequency input modulation in the theta–alpha band when the monkey switched onto the target-in condition (Fig. 4). Previous studies suggested that visual attention; (1) modulates neural firing rates^83–85^, (2) samples visual stimuli rhythmically^41^, and (3) speeds up the sampling frequencies when the visual information is perceptually required to be sampled more frequently^24^. According to these studies, the positive correlation between modulations of neural firing rate and the input frequency (observed in Fig. 4) might be due to the selective allocation of attention toward the stimulus inside the neuron’s RF. These attentional modulations might enhance the temporal resolution of the perceptual processing (neural sampling), thereby improving the neural representation. In line with this, the attenuation of theta–alpha IF modulation in error trials (see Supplementary materials, Figure S1) compared to corresponding IF modulation in hit trials (Fig. 2) might also reflect deteriorations of attentional sampling frequency among the population of MT neurons. We also found that the modulation of theta–alpha frequency is a selective process that emerged among a population of neurons whose RFs overlap with the target stimulus location (comparing the results of the target-in vs. the target-out conditions, Figs. 2, 3). Assessment of the functional significance of the theta–alpha frequencies in relation to the monkey’s behavioral performance revealed a proportional reduction in the magnitude of the theta–alpha IF with increasing the animal’s RTs (Fig. 6A, B). This observation is congruent with a recent study of working memory^26^, showing that higher alpha frequency in the occipital and parietal-occipital regions of the human brain is associated with slower RT. The study suggested that higher-order cortical networks can control the alpha frequency (8–12 Hz) inversely linked to visual cortical excitability to modulate sensory processing in visual neurons. Intriguingly, we further observed that such proportional suppression in theta–alpha IFs of input oscillations could negatively correlate with spiking rates of MT neurons in the local network (Figs. 5C, 6C). Our opposite observations for the variation of the theta–alpha frequencies with MTs’ firing activity at the levels of the single-neuron (Fig. 4) and the neural network (Figs. 5C, 6C) may support the notion that the theta–alpha IF is top-down control, modulating neurons' spike threshold (i.e. the excitability of neurons^80,81^) at each neuronal-scale independently^22^ to effectively improve both of neural representations and routing information to higher-order areas, respectively. In line with this, our data demonstrated that the theta–alpha IF exerts a causal influence on the neuron’s discharging output, probably via changes in the neural spike threshold^22^. Moreover, our further analyses suggested that the frequency of oscillations can play a functional role in the modulation of synchronization between MT neurons, likely allowing sensory-relevant information routes to higher-order areas efficiently^6^.

Our observations for modulation of the theta–alpha frequency are not due to the change in the task difficulty or the task complexity reported by some studies^27,86,87^. Indeed, the task difficulty was the same among two experimental task conditions (i.e. the target-in and the target-out) in this study because there was no difference between physical characteristics of stimuli displayed inside and outside the neurons’ RF (See also the “Methods” for more details). We also showed that the theta–alpha frequency modulation does not result from differences in the SNR of LFPs (i.e. theta–alpha powers) among the two experimental task conditions. Our results implicate flexibility in the frequency of the theta–alpha rhythm^27^, suggesting an operating frequency range within which individual neurons (Fig. 4) and local network neurons (Figs. 5C,D, 6C,D, and S2) function, but it can be modulated at each level distinctly according to changes in task engagement. Additional works are required to unveil which cortical mechanism coordinates different oscillatory frequencies of neighboring neurons into a unified one within a local network, preferentially.

One crucial question is what neurological factors cause rapid fluctuations in the frequency of neural oscillations. Some studies suggested that the activity of specific subsets of inhibitory interneurons can rapidly alter in proportion to the activity of the number of excitatory neurons activated during each oscillation cycle, resulting in cycle-by-cycle changes in the oscillation frequency (interval)^28,88^. It is also thought that recurrent cortical networks play a significant role in adjusting such a rapid balance between excitatory and inhibitory synaptic activities in the local circuit^28^. It is also critical to understand how frequency changes mediating phase shifts within a cortical network modulate the coherency (coupling) among groups of oscillating neurons, allowing the routing of sensory information to associated higher-order areas, efficiently. Our results suggest that de-synchronization of the oscillatory activity (as shown in Fig. 8A for the slow RT trials) might result from increasing input frequencies of neighboring neurons in the local network (as shown in Fig. 6A for the slow RT trials), changing; thereby, the relative phase shift within coherent neurons. These cyclic instantaneous phase shifts can subsequently diminish the efficiency of routing sensory information from the lower to the upper cortical networks. The demonstration of the direct correlative link between theta–alpha frequencies and high-gamma SPCs in fast RT trials (shown in Fig. 8B) is congruent with a recent study, illustrating that the more frequency modulations are, the stronger neural synchronizations^56^.

It has been reported that changes in oscillatory frequency occur in response to the change in; the luminance of visual stimuli^22^, the temporal resolution of visual perception^23^, cognitive demands (e.g. changes in the working memory load)^26,27^, or occur by applying external stimulation like transcranial alternating current stimulation (tACS)^89^. Our finding also provided a crucial step toward understanding how oscillatory frequencies of MT neurons are modulated by deploying spatial attention selectively toward cued stimuli. It is worth noting that although the frequency modulations we observed in this study were small, it is consistent with prior effect size depicting significant frequency modulations on the order of lower than ~ 0.05 Hz^22–26^. Furthermore, due to using a single monkey’s electrophysiological signals to examine potential IF modulations in the visual cortex, additional empirical research is required to test our results' consistency in other monkeys and other animal species.

In sum, we demonstrate that the frequency modulation of cortical rhythmicity in the theta–alpha band is causally associated with changes in; (1) the neural firing rate, (2) high-gamma synchrony and the behavioral reaction time. These observations suggest a link between frequencies of neural oscillations, excitability, synchronization, and behavioral response variability; we postulate a top-down factor (e.g. attention) regulating excitability and synchronization of MT neurons by modulating frequencies of the theta–alpha oscillations to improve neural representation and routing sensory information to higher-order areas effectively.

## Supplementary Information

Supplementary Figures.

## Data Availability

The datasets used and/or analyzed during the current study are available from the corresponding author upon reasonable request and with permission of the German Primate Center – Leibniz Institute for Primate Research (DPZ).
