# Supplementary material for: Frequency modulation of cortical rhythmicity governs behavioral variability, excitability and synchrony of neurons in the visual cortex
Source: Sci Rep. 2022 Dec 3;12:20914. doi: 10.1038/s41598-022-25264-5 (PMC9719482; doi:10.1038/s41598-022-25264-5)
Supplement: Supplementary file 1 — Supplementary Figures. [file 41598_2022_25264_MOESM1_ESM.docx]

Supplementary Materials for

**Frequency modulation of cortical rhythmicity governs behavioral variability, excitability and synchrony of neurons in the visual cortex**

Mohammad Bagher Khamechian^♯^, Mohammad Reza Daliri^♯^

**Supplementary figures**


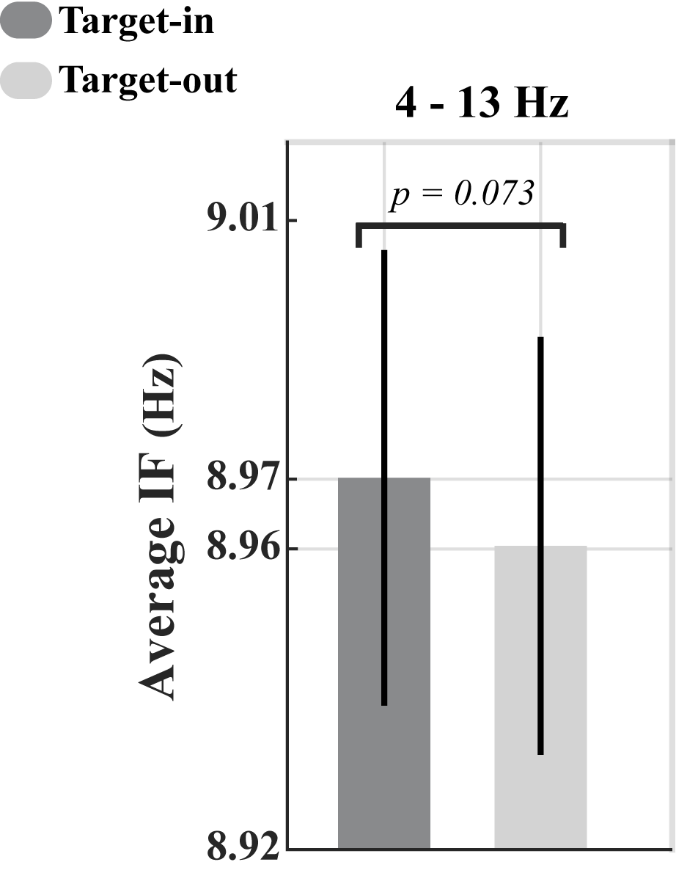


**Figure S1. Modulation of the theta-alpha IF in error trials**. Bars show the average IF of neurons at the conditions of the target-in (the dark bar) and the target-out (the light bar). Since the stimulus presentation in False-alarm trials was shorter than the length of the analysis window used in the main results (I.e. 1500ms, see *Materials and methods*), we only studied the modulation of theta-alpha IF in Miss-trials. The average IF of individual neurons in each condition was calculated using the same algorithm used for Figure 2 (see *Materials and methods*). The figure illustrates that the average IF of neurons' rhythmicity has no significant difference between the target-in and target-out conditions (*p* = 0.073, two-sided sign-test). Notably, before performing the analysis, the number of trials (362 trials) was equalized across the two target conditions.


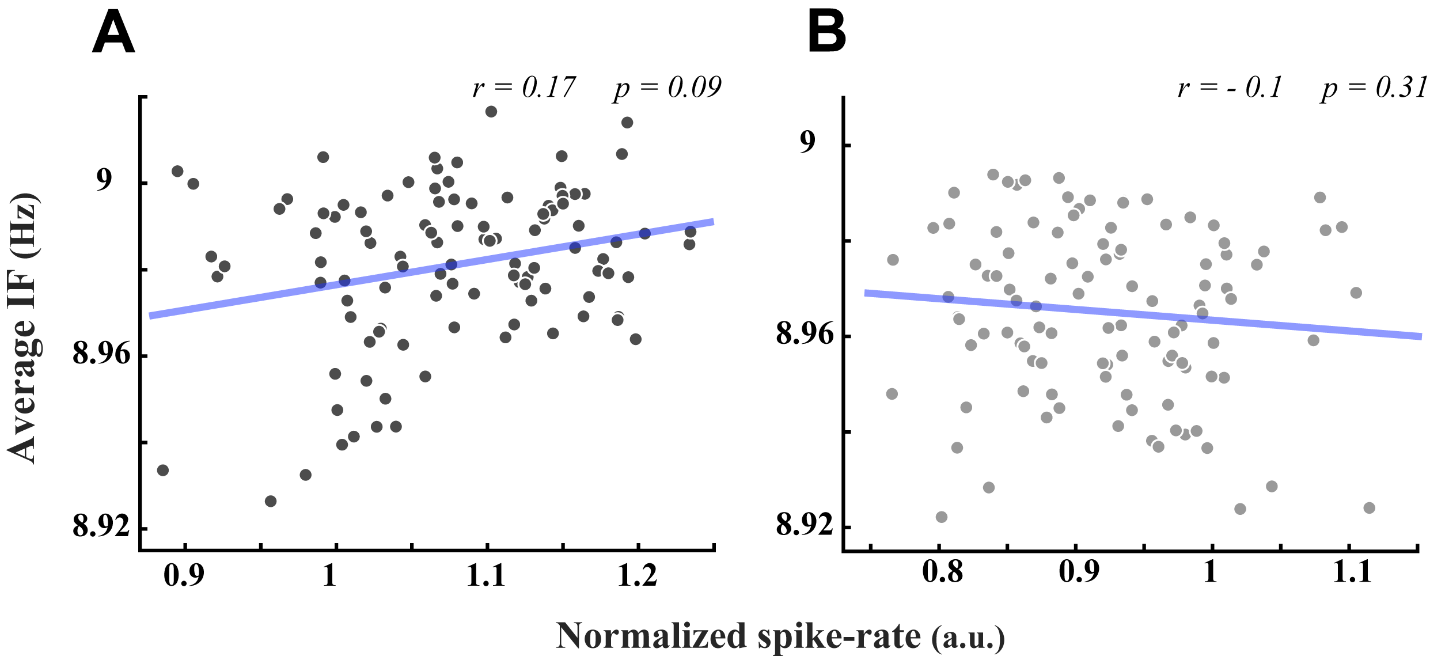


**Figure S2. Correlation between the average frequencies of input oscillations and the neuron’s spiking output.** Circles represent the average theta-alpha IFs versus the normalized spike rate for every single neuron, shown in Figure 4, at the target-in **(A)** and target-out **(B)** conditions. Inset numbers in panels **A-B** represent the correlation magnitude (r, using the Spearman correlation method) and the degree of its significance (p).
